# Effects of Sintering Conditions on Giant Dielectric and Nonlinear Current–Voltage Properties of TiO_2_-Excessive Na_1/2_Y_1/2_Cu_3_Ti_4.1_O_12_ Ceramics

**DOI:** 10.3390/molecules27165311

**Published:** 2022-08-20

**Authors:** Pariwat Saengvong, Jakkree Boonlakhorn, Narong Chanlek, Nutthakritta Phromviyo, Viyada Harnchana, Pairot Moontragoon, Pornjuk Srepusharawoot, Sriprajak Krongsuk, Prasit Thongbai

**Affiliations:** 1 Giant Dielectric and Computational Design Research Group (GD–CDR), Department of Physics, Faculty of Science, Khon Kaen University, Khon Kaen 40002, Thailand; 2 Department of Basic Science and Mathematics, Faculty of Science, Thaksin University, Songkhla 90000, Thailand; 3 Synchrotron Light Research Institute (Public Organization), 111 University Avenue, Muang District, Nakhon Ratchasima 30000, Thailand

**Keywords:** giant/colossal dielectric permittivity, varistor, admittance spectroscopy, impedance spectroscopy, Maxwell–Wagner polarization

## Abstract

The effects of the sintering conditions on the phase compositions, microstructure, electrical properties, and dielectric responses of TiO_2_-excessive Na_1/2_Y_1/2_Cu_3_Ti_4.1_O_12_ ceramics prepared by a solid-state reaction method were investigated. A pure phase of the Na_1/2_Y_1/2_Cu_3_Ti_4.1_O_12_ ceramic was achieved in all sintered ceramics. The mean grain size slightly increased with increasing sintering time (from 1 to 15 h after sintering at 1070 °C) and sintering temperature from 1070 to 1090 °C for 5 h. The primary elements were dispersed in the microstructure. Low dielectric loss tangents (tan δ~0.018–0.022) were obtained. Moreover, the dielectric constant increased from ε′~5396 to 25,565 upon changing the sintering conditions. The lowest tan δ of 0.009 at 1 kHz was obtained. The electrical responses of the semiconducting grain and insulating grain boundary were studied using impedance and admittance spectroscopies. The breakdown voltage and nonlinear coefficient decreased significantly as the sintering temperature and time increased. The presence of Cu^+^, Cu^3+^, and Ti^3+^ was examined using X-ray photoelectron spectroscopy, confirming the formation of semiconducting grains. The dielectric and electrical properties were described using Maxwell–Wagner relaxation, based on the internal barrier layer capacitor model.

## 1. Introduction

Dielectric materials are among the most interesting materials for use in electronic devices such as capacitors and high-energy storage devices [1,2]. CaCu_3_Ti_4_O_12_ (CCTO) has been extensively studied because CCTO can exhibit a high dielectric constant (ε′ > 10^4^) [3,4,5,6,7,8]. Furthermore, CCTO ceramic is not a ferroelectric material. The observed large ε′ value was stable with respect to temperature and frequency. However, the dielectric loss tangent remains too high (tan δ > 0.1) and cannot be applied to electronic devices. Many years ago, researchers attempted to improve the dielectric properties of CCTO by increasing ε′, reducing tan δ, and increasing the efficiency of dielectric properties for stability with temperature and frequency [6,7,9,10]. It is believed that the origin of the dielectric properties of CCTO arises from its microstructure, consisting of an insulating grain boundary (i-GBs) and a semiconducting grain (semi-G). This microstructure is called an internal barrier layer capacitor (IBLC) structure [3,11].

In the IBLC model, tan δ can be reduced by increasing the grain boundary resistance (R_gb_) [12,13]. In 2006, Y. Lin et al. reduced the tan δ in CCTO from 0.07 to 0.03 by adding composite TiO_2_ into samples [9]. When the R_gb_ was analyzed using impedance spectroscopy, it appeared that R_gb_ increased with increasing TiO_2_ contents in CCTO ceramics. Moreover, the breakdown electric field (E_b_) of the nonlinear J-E characteristics increased with increasing TiO_2_ contents, indicating that R_gb_ can be increased by adding TiO_2_ to CCTO ceramics, resulting in a decrease in tan δ, which corresponds to the IBCL model [9,14].

The optimized sintering condition is one technique that has been used to improve dielectric properties, which can modify the microstructure of ceramics. The effect of sintering temperature results in grain size changes and i-GBs. Generally, ε′ can be increased by increasing the temperature and duration of sintering in ACu_3_Ti_4_O_12_ ceramics [11,15,16,17].

ACu_3_Ti_4_O_12_ is a dielectric material group that has received extensive attention because of its interesting dielectric properties. CaCu_3_Ti_4_O_12_ (CCTO) [4,5], CdCu_3_Ti_4_O_12_ (CdCTO) [18,19], Na_1/2_Sm_1/2_Cu_3_Ti_4_O_12_ (NSmCTO) [20], Na_1/2_Y_1/2_Cu_3_Ti_4_O_12_ (NYCTO) [16,21,22,23], Na_1/2_La_1/2_Cu_3_Ti_4_O_12_ (NLCTO) [24,25], Y_2/3_Cu_3_Ti_4_O_12_ (YCTO) [26,27], La_2/3_Cu_3_Ti_4_O_12_ (LCTO) [28], Bi_2/3_Cu_3_Ti_4_O_12_ (BCTO) [29,30,31,32], and Na_1/2_Bi_1/2_Cu_3_Ti_4_O_12_ (NBCTO) [17,33,34] were dielectric materials in the ACu_3_Ti_4_O_12_ oxides group. These materials have perovskite structures and often have reported excellent dielectric properties. However, these materials still have limitations under many conditions for application in electronic devices. The dielectric properties of CCTO and NYCTO ceramics can be significantly improved by the addition of excess TiO_2_ [9,21].

Most recently, the tan δ value of Na_1/2_Y_1/2_Cu_3_Ti_4.1_O_12_ can be significantly reduced (tan δ < 0.1), while the ε′ value is still larger than 10^4^. The improved dielectric properties were attributed to the enhanced electrical properties of the i-GBs due to the excessive TiO_2_-rich phase boundaries, which are also associated with the oxygen content along the i-GBs. Generally, the giant dielectric properties of CCTO-based materials are closely related to sintering conditions [5,35,36]. Thus, the giant dielectric properties can be optimized by tuning the sintering conditions. It is hypothesized that the dielectric properties of Na_1/2_Y_1/2_Cu_3_Ti_4.1_O_12_ can be further improved by varying the sintering conditions. The optimization of fabrication conditions is one of the most important topics for developing materials used in electronic devices. This is the motivation of this work. Hence, the aim of this study was to investigate the effect of sintering conditions on the dielectric properties of Na_1/2_Y_1/2_Cu_3_Ti_4.1_O_12_ to optimize the dielectric properties.

In this study, the effects of sintering conditions on the dielectric and electrical properties of Na_1/2_Y_1/2_Cu_3_Ti_4.1_O_12_ ceramics were investigated. The phase composition and microstructure were analyzed. Significant improvements in the dielectric and electrical behaviors with respect to ε′ and tan δ were achieved. The origin of the observed dielectric and electrical properties is described.

## 2. Experimental Details

Na_1/2_Y_1/2_Cu_3_Ti_4.1_O_12_ powder was prepared using the solid-state reaction method (SSR). The starting materials were Na_2_CO_3_ (Sigma-Aldrich, St. Louis, MO, USA, 99.9%), Y_2_O_3_ (CERAC, 99.99%), CuO (Sigma-Aldrich, St. Louis, MO, USA, 99.9%), and TiO_2_ (Sigma-Aldrich, St. Louis, MO, USA, 99.9%). The powders were mixed using a ball milling method for 24 h. The powder was calcined at 1000 °C in air for 10 h at heating and cooling rates of 5 °C/min. The calcined powder was finely crushed and compressed into pellets for sintering. The first set of pellets was sintered at 1070 °C for 1, 5, and 15 h. The second set of pellets was sintered at 1080 and 1090 °C for 5 h. The samples produced under these conditions are referred to as 1NYCTTiO, 2NYCTTiO, 3NYCTTiO, 4NYCTTiO, and 5NYCTTiO, respectively.

The phase compositions were analyzed by X-ray diffraction (XRD, PANalytical, EMPYREAN). The XRD patterns were measured in the 2θ range of 25–70°, and the lattice parameters were calculated using the Rietveld technique. The surfaces of the ceramics were characterized by scanning electron microscopy (SEM, SEC, SNE-4500 M). The element distributions were analyzed using a mapping technique, and field emission scanning electron microscopy (FIB-FESEM) was used for SEM mapping. X-ray photoemission spectroscopy (XPS, PHI5000 Versarobe II, ULVAC-PHI) was used to analyze the oxidation states of the Na_1/2_Y_1/2_Cu_3_Ti_4.1_O_12_ ceramics. The densities of the sintered samples were measured. 

To determine the dielectric and electrical properties, the as-sintered ceramics were polished to a smooth surface. Next, parallel-plate electrodes were made by painting Ag paste on both sides of the smooth surfaces. Then, the sample with top and bottom electrodes was heated in air at 600 °C for 30 min. The area of the top and bottom electrodes was 2.83 cm^2^. The capacitance (C_p_) and tan δ were measured in the frequency range of 10^2^ to 10^6^ Hz at room temperature using an impedance analyzer (KEYSIGHT E4990A). The amplitude of the ac field was 0.5 V. In addition, the dielectric behavior was studied in the temperature range of −60 to 210 °C. Finally, the nonlinear electrical (I–V) properties were diagnosed using a high-voltage measurement unit (Keithley 247 model).

The ε′ value was calculated by the equation:

(1) $$\varepsilon^{\prime }=\frac{C_{p}t}{\varepsilon_{0}S}$$

where ε_0_ is the permittivity of free space and S and t are the electrode area and sample thickness, respectively. The complex dielectric permittivity ($\varepsilon^{*}$) was used to calculate the complex impedance (Z*) using Equation (2).

(2) $$\varepsilon^{*}=\varepsilon^{\prime }-i\;\varepsilon^{\prime \prime }={\left({i\omega C}_{0}Z^{*}\right)}^{-1}={\left[{i\omega C}_{0}\left(Z^{\prime }-i\;Z^{\prime \prime }\right)\right]}^{-1}$$

where $Z^{\prime }$ and $Z^{\prime \prime }$ are the real part and imaginary part of Z*, respectively, $\varepsilon^{\prime }$ and $\varepsilon^{\prime \prime }$ are the real and imaginary parts of $\varepsilon^{*}$, $C_{0}=\varepsilon_{0}A/d$ is the empty cell capacitance, $\tan\delta =\varepsilon^{\prime \prime }/\varepsilon^{\prime }$, and ω is the angular frequency (ω = 2πf).

## 3. Results and Discussion

**Figure 1:**
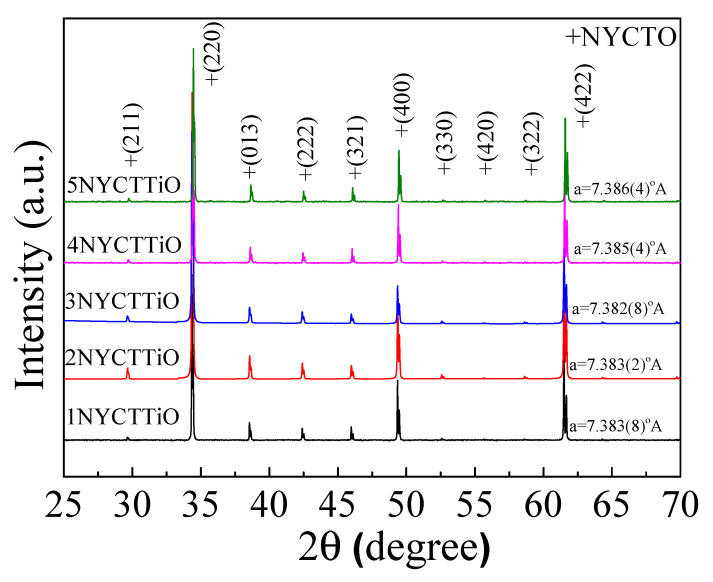
XRD patterns of the Na_1/2_Y_1/2_Cu_3_Ti_4.1_O_12_ ceramics sintered at 1070 °C for 1, 5, and 15 h and sintered at 1080 and 1090 °C for 5 h.

The XRD characteristics of the sintered Na_1/2_Y_1/2_Cu_3_Ti_4.1_O_12_ ceramics were analyzed. Accordingly, the lattice parameters were calculated using the Rietveld technique. Figure 1 shows the XRD patterns of all samples, showing the pure phase (not detecting the second phase and not detecting the TiO_2_ phase) with the perovskite structure (JCPDS 75–2188). The lattice parameters of 1NYCTTiO, 2NYCTTiO, 3NYCTTiO, 4NYCTTiO, and 5NYCTTiO were 7.383(8), 7.383(2), 7.382(8), 7.385(4), and 7.386(4) Å, respectively. The sintering conditions did not affect the lattice parameters of the Na_1/2_Y_1/2_Cu_3_Ti_4.1_O_12_ ceramics. Moreover, the XRD patterns and lattice parameters were comparable to the XRD spectra that were reported for Na_1/2_Y_1/2_Cu_3_Ti_4_O_12_ (NYCTO) ceramics [16,21,22,23].

**Figure 2:**
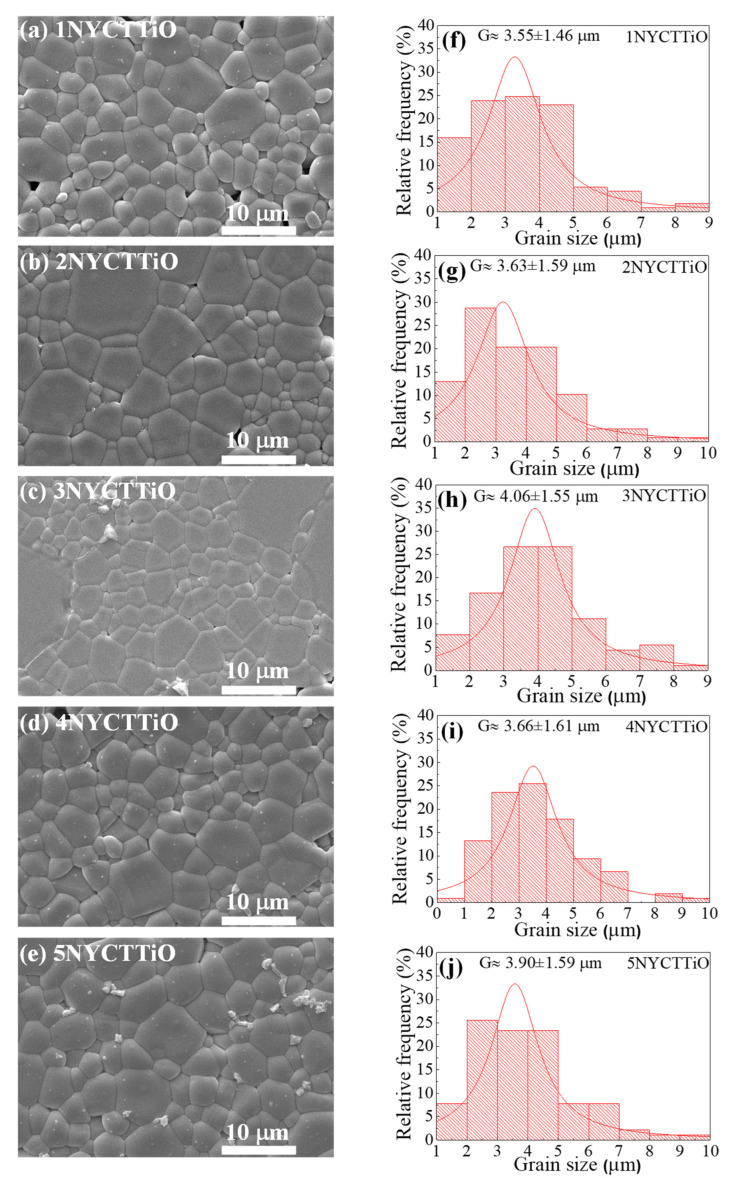
SEM images of polished surfaces of Na_1/2_Y_1/2_Cu_3_Ti_4.1_O_12_ ceramics sintered at (**a**) 1070 °C for 1 h, (**b**) 1070 °C for 5 h, (**c**) 1070 °C for 15 h, (**d**) 1080 °C for 5 h, and (**e**) 1090 °C for 5 h and grain size distributions with sintering at (**f**) 1070 °C for 1 h, (**g**) 1070 °C for 5 h, (**h**) 1070 °C for 15 h, (**i**) 1080 °C for 5 h, and (**j**) 1090 °C for 5 h.

Figure 2a–e show the morphologies of Na_1/2_Y_1/2_Cu_3_Ti_4.1_O_12_ ceramics sintered under different conditions using the SEM technique. The SEM images clearly show the grains and GBs. A small number of pores can be observed. The grain-size distributions are shown in Figure 2f–j. The mean grain sizes of the 1NYCTTiO, 2NYCTTiO, 3NYCTTiO, 4NYCTTiO, and 5NYCTTiO samples were 3.55 ± 1.46, 3.63 ± 1.59, 4.06 ± 1.55, 3.66 ± 1.61, and 3.90 ± 1.59 μm, respectively. The average grain size increased slightly with increasing temperature and time in the sintering process, which may have been caused by the enhanced diffusion of ions [6,7]. The densities of the samples were 96.34, 97.40, 97.67, 95.61, and 96.90%, respectively.

**Figure 3:**
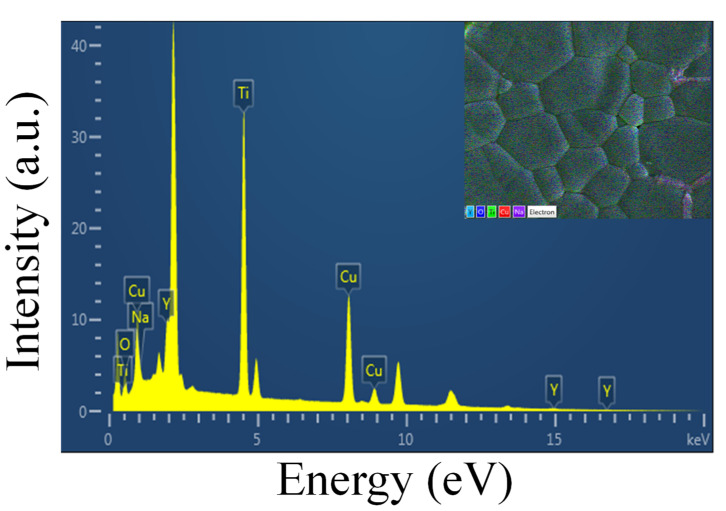
EDS spectrum of Na_1/2_Y_1/2_Cu_3_Ti_4.1_O_12_ ceramic sintered at 1070 °C for 15 h.

**Figure 4:**
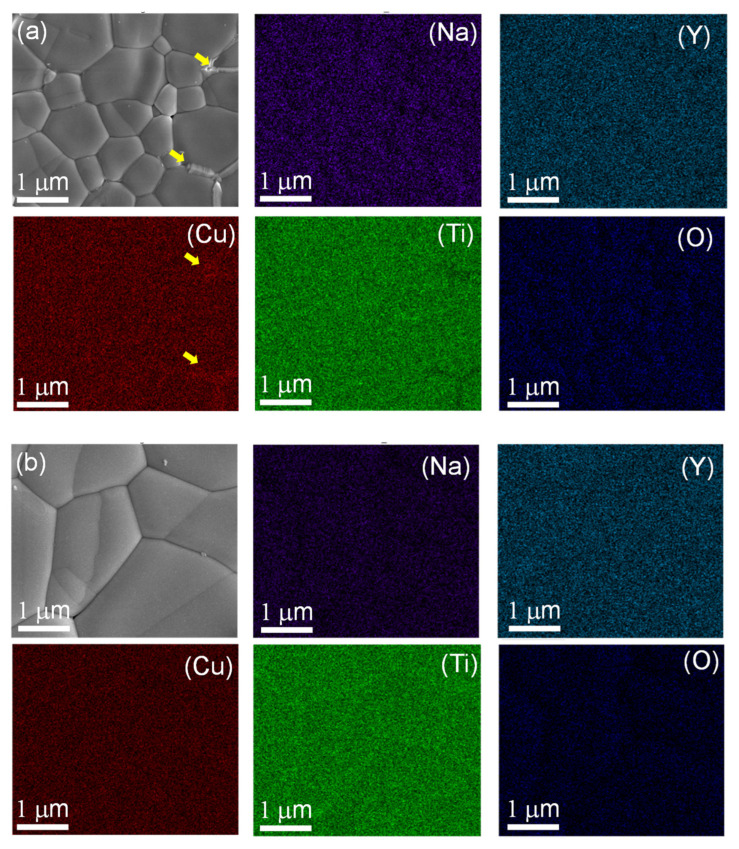
SEM mapping images of Na, Y, Cu, Ti, and O considering the amount of Cu (**a**) and Ti (**b**) at the grain boundary of the Na_1/2_Y_1/2_Cu_3_Ti_4.1_O_12_ ceramic sintered at 1070 °C for 15 h.

The elemental composition of the Na_1/2_Y_1/2_Cu_3_Ti_4.1_O_12_ ceramic sintered at 1070 °C for 15 h was studied using EDS. The EDS spectrum shown in Figure 3 can be used to observe the major elements consisting of Na, Y, Cu, Ti, and O peaks to confirm the elemental composition of the ceramics. The mapping technique was used to study the elemental distribution, as shown in Figure 4a,b. All elements were homogeneously dispersed inside the grains. Figure 4a shows the distribution of elements; all elements were well-distributed throughout the sample. However, segregation of Cu was observed around the GB to detect CuO, which may have been caused by the effect of sintering temperature and increasing Ti concentration. The concentration of excess Ti may enter into the Cu sites in the structure, causing the segregation of CuO at the GB during the temperature sintering process [9,10,11]. Moreover, when examining the distribution of elements at the GB in the area without CuO, Ti element was detected at the GB, indicating that excess Ti tends to unite at the GB, as shown in Figure 4b. Generally, TiO_2_ and CuO have electrical insulating properties; detecting this at the GB may result in an increase in resistance at the GB, which is an important factor for reducing tan δ in perovskite dielectric materials [10].

**Figure 5:**
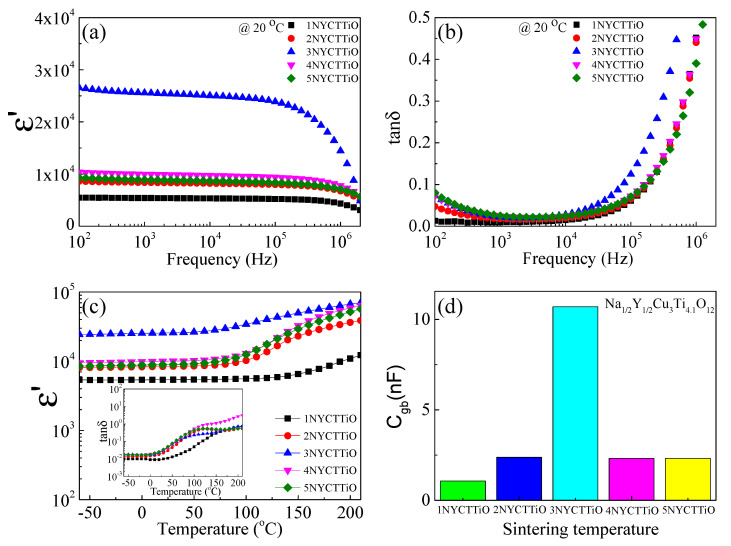
(**a**) Dielectric constant (ε′) and (**b**) dielectric loss tangent (tan δ) at room temperature (20 °C) as a function of frequency for Na_1/2_Y_1/2_Cu_3_Ti_4.1_O_12_ ceramics sintered under different conditions. (**c**) Dielectric constant (ε′) and (inset) dielectric loss tangent (tan δ) as a function of temperature in the range of −60 to 210 °C. (**d**) Grain-boundary capacitance (C_gb_).

Depending on the frequency shown in Figure 5a, the ε′ values at 1 kHz and 20 °C for the 1NYCTTiO, 2NYCTTiO, 3NYCTTiO, 4NYCTTiO, and 5NYCTTiO samples were 5396, 8370, 25,565, 9972, and 8896, respectively. The ε′ values tended to increase with increasing sintering duration and slightly increased with increasing sintering temperature, which might be a result of the increasing grain size when this result was considered using the IBLC model [6,15,16,17,18]. It was also observed that ε′ was a relatively stable frequency. However, at a high frequency (>10^5^ Hz), ε′ rapidly dropped because of the dielectric relaxation process. Moreover, all the samples had a low tan δ (<0.05) over the frequency range of 70–100 kHz. tan δ slightly changed with increasing sintering duration and sintering temperature. The tan δ values of the samples were 0.009, 0.018, 0.022, 0.024, and 0.025, respectively, as observed in Figure 5b. However, tan δ increased rapidly at frequencies higher than 10^5^ Hz. This is the dielectric relaxation response, which is related to the fast ε′ drop at high frequencies. These behaviors are similar to the dielectric behaviors that occur in CCTO and NYCTO ceramics [1,2,3,4,5,6,7,8]. The dielectric relaxation at high frequencies was caused by the fact that the electric dipole cannot complete the polarization process because the relaxation time is too small.

Figure 5c shows the dependence of ε′ and tan δ on the temperature. The ε′ and tan δ values of the 2NYCTTiO, 4NYCTTiO, and 5NYCTTiO samples were greatly increased when the temperature was above 90 °C. These dielectric responses were caused by the electric charge having high energy (maybe more than the potential energy at the GB) and being easier to move at a high temperature. As a result, the polarization process occurs more easily and the electrical conductivity increases [19,20,21,22]. However, the ε′ values of the 1NYCTTiO and 3NYCTTiO samples were relatively stable with temperature, indicating that the sintering condition for ε′ stability with temperature was successfully achieved. As shown in Figure 5d, the GB capacitance (C_gb_) values were calculated in the temperature range of 150–200 °C using the electric modulus (M*) technique. The C_gb_ values of the 1NYCTTiO, 2NYCTTiO, 3NYCTTiO, 4NYCTTiO, 5NYCTTiO samples were 1.07, 2.38, 10.7, 2.32, and 2.32 nF, respectively. The C_gb_ values did not change with increasing sintering temperatures due to the small change in the sintering temperature. However, C_gb_ tended to increase with increasing sintering duration, which corresponds to an increase in ε′. This may be the major factor causing the ε′ rise when using the IBLC model [6]. In this study, excellent dielectric properties were obtained for the 3NYCTTiO samples. 

The excellent dielectric properties of NYCTO are attributed to the electrically the heterogeneous microstructure of the materials. These microstructures consist of i-GBs (high resistance) and semi-Gs (low resistance). The electrical circuit model explained the electrical conductivity of the materials, which consisted of a capacitor (C) and a resistor (R) connected in series (RC circuits). Thus, impedance spectroscopy was used to analyze the electrical properties of the ceramic materials. Normally, the grain resistance (R_g_) and GB resistance (R_gb_) are obtained by the small semicircle (or nonzero intercept) and large semicircle observed from Z* plots, respectively. Moreover, the grain capacitance (C_g_) and GB capacitance (C_gb_) can be calculated using impedance spectroscopy [2,23]. Z^*^ is calculated using Equation (3).

(3) $$Z^{*}=Z^{\prime }-iZ^{\prime \prime }=\frac{1}{R_{g}^{-1}+{i\omega C}_{g}}+\frac{1}{R_{\mathrm{gb}}^{-1}+{i\omega C}_{\mathrm{gb}}}$$

**Figure 6:**
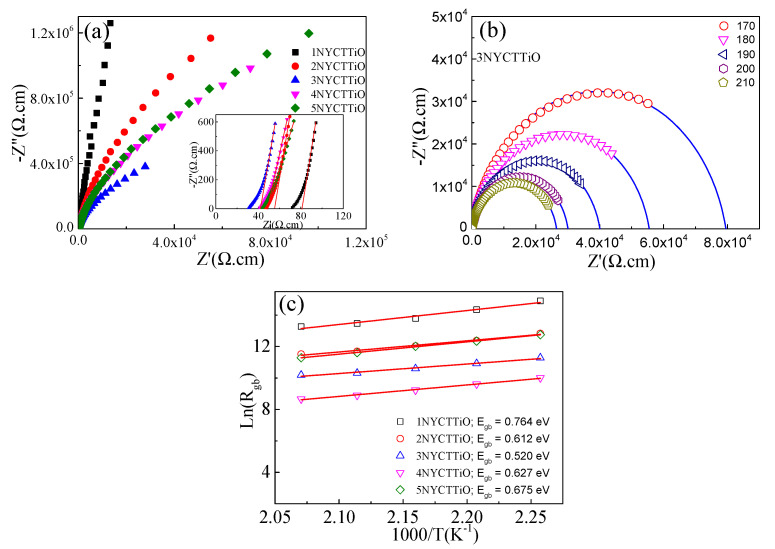
(**a**) Impedance complex plane plot at 20 °C and inset non-zero intercept of Na_1/2_Y_1/2_Cu_3_Ti_4.1_O_12_ ceramics sintered at 1070 °C for 1, 5, and 15 h and sintered at 1080 and 1090 °C for 5h. (**b**) Impedance complex plane plot in the temperature range of 170–210 °C for the sample sintered at 1070 °C for 15 h. (**c**) Arrhenius plot of the grain boundary conductivity (R_gb_).

Figure 6a shows the impedance complex plane (Z*) plots of the 1NYCTTiO, 2NYCTTiO, 3NYCTTiO, 4NYCTTiO, and 5NYCTTiO samples. The large semicircle tended to decrease with increasing sintering duration and slightly decreased with increasing sintering temperature, indicating that R_gb_ tended to decrease with increasing sintering duration and sintering temperature. Moreover, the R_g_ tended to decrease with increasing sintering duration (R_g_ was trivially changed with increasing sintering temperature) when observed from the non-zero intercept in the inset of Figure 6a. The effects of changing the R_g_ and R_gb_ on the dielectric properties were investigated.

To calculate the activation at the GB (E_gb_), R_gb_ was obtained by fitting the Z* data with the IBLC model at various temperatures. For the IBLC structure of CCTO-based ceramics, the microstructure consisted of i-GBs and semi-G [3]. Generally, the C_gb_ values of the ceramics were much higher than their C_g_ values (C_gb_ >> C_g_). Therefore, Equation (3) can be modified to create a fitting model as follows [9]:

(4) $$Z^{*}=R_{g}+\frac{R_{\mathrm{gb}}}{1+{\left({i\omega R}_{\mathrm{gb}}C_{\mathrm{gb}}\right)}^{\alpha }}$$

where α is a constant (0<α≤1). The R_gb_ values were obtained by fitting the experimental data of Z* in the temperature range of 170–210 °C. Figure 6b shows the fitted results of Z* plots for the 3NYCTTiO sample for calculating the E_gb_ value. The other samples were fitted using the same process (data not shown). Next, the E_gb_ values were calculated using the Arrhenius law:

(5) $$R_{\mathrm{gb}}=R_{0}\exp\left(\frac{E_{\mathrm{gb}}}{k_{B}T}\right)$$

where k_B_ is the Boltzmann constant, *T* is the temperature (Kelvins), and R_0_ is a pre-exponential constant term. As shown in Figure 6c, the E_gb_ values of 1NYCTTiO, 2NYCTTiO, 3NYCTTiO, 4NYCTTiO, and 5NYCTTiO were 0.764, 0.612, 0.520, 0.627, and 0.675 eV, respectively. The E_gb_ values tended to decrease with increasing sintering durations, but the E_gb_ values trivially changed with increasing sintering temperatures, similar to R_gb_. The E_gb_ values of all samples were close to those reported for CCTO and NYCTO ceramics (≈0.6 eV), confirming that the GBs of the samples were electrically insulating [1,6,24,25]. Furthermore, the decrease in the E_gb_ values might be caused by an increase in the number of oxygen vacancies.

**Figure 7:**
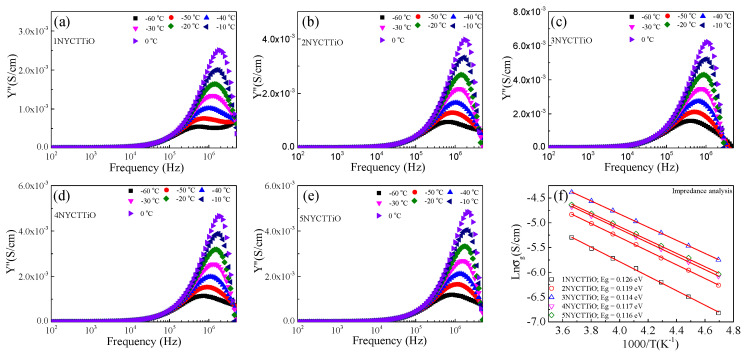
Admittance imaginary (Y″) as a function of the frequency plot in the temperature range of −60–0 °C for Na_1/2_Y_1/2_Cu_3_Ti_4.1_O_12_ ceramics sintered at (**a**) 1070 °C for 1 h, (**b**) 1070 °C for 5 h, (**c**) 1070 °C for 15 h, (**d**) 1080 °C for 5 h, and (**e**) 1090 °C for 5 h. (**f**) Arrhenius plot of grain conductivity (σ_g_).

To study the dielectric properties of Na_1/2_Y_1/2_Cu_3_Ti_4.1_O_12_ and other ACu_3_Ti_4_O_12_ compounds, the conductivity of the semi-Gs was considered to be an important factor. Generally, R_g_ is calculated from the nonzero intercept on the Z’-axis in the Z* plot. The conductivity of the semi-Gs was confirmed by calculating the E_g_ value (activation energy in the grain). In this study, E_g_ was calculated using admittance spectroscopy (Y*). According to the IBLC model, for the equivalent circuit (RC), when R_g_ << R_gb_ and C_g_ << C_gb_, the electrical conductivity can be analyzed using Equation (6):

(6) $$Y^{*}=\frac{\left(1-\omega^{2}\tau_{g}\tau_{\mathrm{gb}}+{i\omega \tau }_{\mathrm{gb}}\right)\left(R_{\mathrm{gb}}^{-1}\right)}{1+i\omega \tau }$$

where $Y^{*}=\left(1+{i\omega R}_{g}C_{g}\right)/R_{g}$ and $Y^{*}=\left(1+{i\omega R}_{\mathrm{gb}}C_{\mathrm{gb}}\right)/R_{\mathrm{gb}}$. $\tau =R_{g}C_{\mathrm{gb}}$, $\tau_{g}=R_{g}C_{g}$, and $\tau_{\mathrm{gb}}=R_{\mathrm{gb}}C_{\mathrm{gb}}$. When using Equation (6), this equation can be reduced to $R_{g}=1/2Y_{\max}^{\prime \prime }$, where $Y_{\max}^{\prime \prime }$ is the maximum value at $Y^{\prime \prime }$-peak. The $Y_{\max}^{\prime \prime }$ values of the 1NYCTTiO, 2NYCTTiO, 3NYCTTiO, 4NYCTTiO, and 5NYCTTiO samples are shown in Figure 7a–e. In addition, the E_g_ values were calculated using the Arrhenius law:

(7) $$\sigma_{g}=\sigma_{0}e^{\left(\frac{-E_{g}}{k_{B}T}\right)}$$

where E_g_ is the activation energy for the conductivity in the grain. The E_g_ values were calculated in the temperature range from −60 to 0 °C, as shown in Figure 7f. The E_g_ values of 1NYCTTiO, 2NYCTTiO, 3NYCTTiO, 4NYCTTiO, and 5NYCTTiO were 0.126, 0.119, 0.114, 0.117, and 0.116 eV, respectively. The E_g_ values were close to those reported for NYCTO ceramics, indicating that the grain was a semiconductor [5,6,8,26,27]. Moreover, E_g_ tends to decrease slightly with increasing sintering duration (E_g_ is trivially changed with increasing sintering temperatures), and the sintering duration corresponds to a decrease in R_g._ The reductions in E_g_ and R_g_ correspond to an increase in ε′, which is an important factor for explaining the dielectric properties of Na_1/2_Y_1/2_Cu_3_Ti_4.1_O_12_ ceramics. The relationship between the dielectric permittivity and the electrical conductivity of the semi-Gs and i-GBs is presented in Table 1.

**Figure 8:**
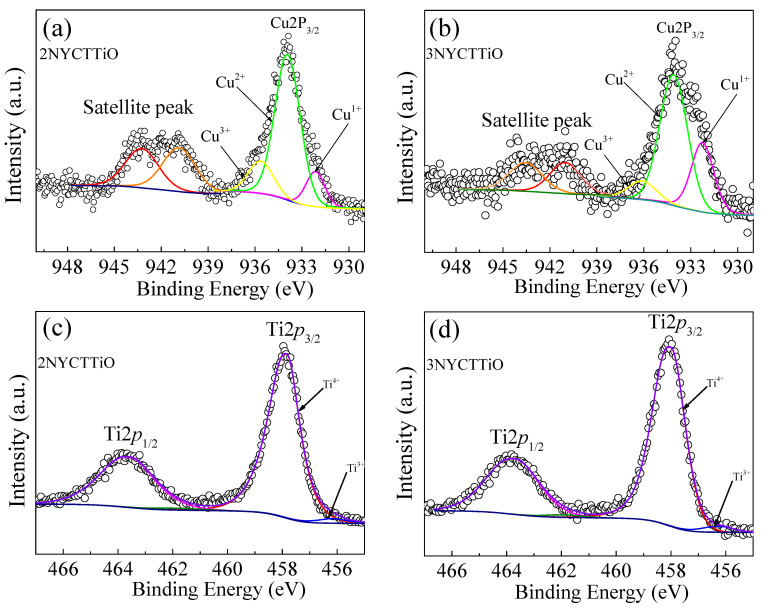
(**a**,**b**) XPS spectra of Cu2p and (**c**,**d**) XPS spectra of Ti2p_3/2_ for Na_1/2_Y_1/2_Cu_3_Ti_4.1_O_12_ ceramics sintered at 1070 °C for 5 and 15 h.

Normally, the grains of ACu_3_Ti_4_O_12_ ceramics are semiconducting. Thus, studying the electrical properties of grains plays an important role in changing the dielectric properties of ceramic materials. The oxidation states of the Ti and Cu ions in the grains were investigated using XPS. Figure 8a–d show the electron hopping between Cu^+^ ↔ Cu^2+^, Cu^2+^ ↔ Cu^3+^, and Ti^3+^ ↔ Ti^4+^ in the 2NYCTTiO and 3NYCTTiO samples. The peaks of Cu2p_3/2_ were fitted using Gaussian–Lorentzian models, as shown in Figure 8a,b, and the peaks of Cu^+^, Cu^2+^, and Cu^3+^ were observed at the mean binding energies of approximately ≈932.13–932.25, 933.92–934.09, and 935.63–936.10 eV, respectively. The calculated Cu^+^/Cu^2+^ of the 2NYCTTiO and 3NYCTTiO samples were 18.34% and 45.62%. The Cu^+^/Cu^2+^ ratio tends to increase with increasing sintering durations. Two peaks of Ti 2*p*_3/2_ are shown in Figure 8c,d. The Ti^3+^/Ti^4+^ ratios were 3.33% and 3.18%, which almost did not change with increasing sintering durations, indicating that the effect of sintering was not affected by electron hopping between Ti^3+^ ↔ Ti^4+^. Thus, the electrical properties of the grains may result from electron hopping between Cu^+^ ↔ Cu^2+^. Generally, the electrical conductivity depends on the concentration of free charges and the mobility of the charge carriers in the material. ACu_3_Ti_4_O_12_ ceramics often lose oxygen during sintering, resulting in oxygen vacancies and free electrons [28,29]. Accordingly, increasing the Cu^+^/Cu^2+^ ratio indicates an increase in the electrical conductivity of the grains. The increasing electrical conductivity within the grains was related to a decrease in R_g_, which may be the major cause of the ε′ increase, which can be described based on the IBLC model [30]. 

**Figure 9:**
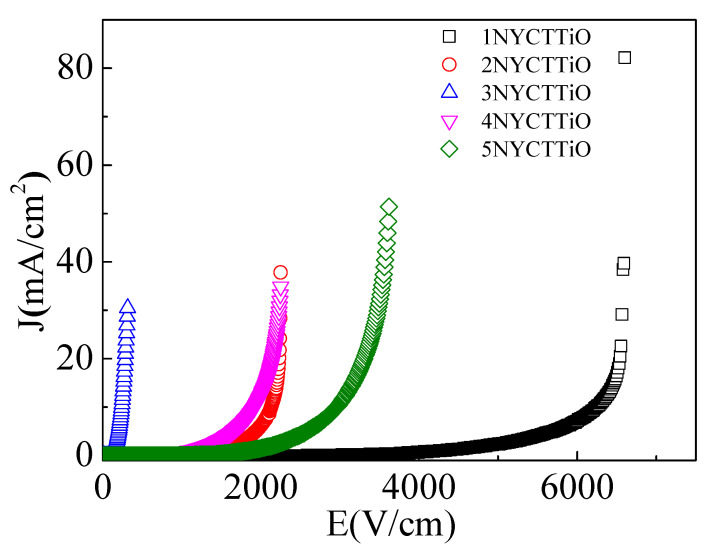
Nonlinear J-E characteristics of Na_1/2_Y_1/2_Cu_3_Ti_4.1_O_12_ ceramics sintered at 1070 °C for 1, 5, and 15 h and sintered at 1080 and 1090 °C for 5 h.

The non-ohmic characteristics of ACu_3_Ti_4_O_12_ ceramics have been extensively studied to explain their electrical behavior in the R_gb_ of materials. The effect of sintering duration on the nonlinear current density–electric field (J–E) characteristics of the Na_1/2_Y_1/2_Cu_3_Ti_4.1_O_12_ ceramics is shown in Figure 9. The E_b_ and the nonlinear coefficient (α) can be calculated. Both values can be calculated using the equation of varistor characteristics (I = KV^α^), where K is the constant related to the resistance of the material. The α values were calculated in the current density (J) range of 1–10 mA, and the E_b_ values were obtained at *J* = 1 mA. The α and E_b_ values of the 1NYCTTiO, 2NYCTTiO, 3NYCTTiO, 4NYCTTiO, and 5NYCTTiO samples were 6.1, 5.7, 4.1, 4.2, and 4.7 and 4251.5, 1426.50, 138.2, 1101.2, and 1825.4 V/cm, respectively. The E_b_ and α values tended to decrease with increasing temperature durations, which is similar to the electrical behavior of NYCTO ceramics [2,6]. Both values support R_gb_ and E_gb,_ which decreased with increasing temperatures. With respect to the sintering temperature effect, E_b_ was slightly decreased in the 4NYCTTiO sample (sintered at 1080 °C for 5 h) but increased in the 5NYCTTiO sample (sintered at 1090 °C for 5 h) when compared with the 2NYCTTiO sample (sintered at 1070 °C for 5 h). In the 4NYCTTiO sample, the slight decrease in the E_b_ values may have been caused by the enhancement in the oxygen vacancy concentration at the i-GBs in the sintering temperature mechanism. However, the increased E_b_ values in the 5NYCTTiO sample may have been caused by an increase in the CuO content at the GB in the high-temperature sintering process.

The non-ohmic properties of NYCTO may be related to the oxygen concentration. Normally, R_gb_ can be increased by increasing the Ti and oxygen contents, which is a method for changing the non-ohmic properties of NYCTO ceramics [10,24]. However, the oxygen content was reduced by increasing the temperature. Therefore, the electrical resistance at the GBs of the Na_1/2_Y_1/2_Cu_3_Ti_4.1_O_12_ ceramics might be decreased by this effect. However, the effect of changing the microstructure on non-ohmic properties should be considered.

## 4. Conclusions

The average grain sizes of the Na_1/2_Y_1/2_Cu_3_Ti_4.1_O_12_ ceramics sintered under different conditions tended to increase slightly with sintering durations and temperatures. All major elements were detected in the microstructure, and the segregation of Cu and Ti was observed along the grain boundaries. The E_b_ and α values tended to decrease with increasing sintering durations. The Na_1/2_Y_1/2_Cu_3_Ti_4.1_O_12_ ceramics sintered at 1070 °C for 1, 5, and 15 h and sintered at 1080 and 1090 °C for 5 h presented low and slightly changed tan δ values (0.009-0.025). The giant ε′ increased with increasing sintering durations and sintering temperatures (5396–25,565). The impedance spectroscopy confirmed the IBLC structure in the Na_1/2_Y_1/2_Cu_3_Ti_4.1_O_12_ ceramics, consisting of semi-Gs and i-GBs. The origin of n-type semiconducting grains was considered using the XPS results, and the existence of Cu^+^, Cu^3+^, and Ti^3+^ was detected. This result may have been caused by oxygen loss from the sintering duration and temperature process. The overall giant dielectric permittivity and electrical properties were described using Maxwell–Wagner relaxation polarization.

## Figures and Tables

**Table 1:** ε′ at 1 kHz and 20 °C, R_g_ at 20 °C, and calculated E_g_, E_gb,_ and C_gb_ of Na_1/2_Y_1/2_Cu_3_Ti_4.1_O_12_ sintered at 1070 °C for 1, 5, and 15 h and sintered at 1080 and 1090 °C for 5 h.

Sample	R_g_ (Ω)	E_g_ (eV)	E_gb_ (eV)	C_gb_ (nF)	ε’
1NYCTTiO	83	0.126	0.764	1.07	5396
2NYCTTiO	54	0.119	0.612	2.38	8370
3NYCTTiO	41	0.114	0.520	10.7	25,565
4NYCTTiO	48	0.117	0.627	2.32	9972
5NYCTTiO	49	0.116	0.675	2.32	8896

## Data Availability

The data presented in this study are available in article.

## Sample Availability

Samples of the compounds are available from the authors.
